# Comparative transcriptome and proteome profiling of two *Citrus sinensis* cultivars during fruit development and ripening

**DOI:** 10.1186/s12864-017-4366-2

**Published:** 2017-12-21

**Authors:** Jian-hui Wang, Jian-jun Liu, Ke-ling Chen, Hong-wen Li, Jian He, Bin Guan, Li He

**Affiliations:** 1 Horticulture Institute, Sichuan Academy of Agricultural Sciences, Chengdu, 610066 China; 2 State-Sichuan Joint Engineering Laboratory of Citrus Breeding and Cultivation, Chengdu, 610066 China; 3 The Key Laboratory of Horticultural Crops Biology and Germplasm Enhancement in Southwest Regions, Ministry of Agriculture, Chengdu, 610066 China

**Keywords:** *Citrus sinensis*, Blood orange, Navel orange, Fruit quality, Transcriptome, Proteome

## Abstract

**Background:**

Transcriptome and proteome analyses on fruit pulp from the blood orange ‘Zaohong’ and the navel orange ‘twenty-first century’ were performed to study *Citrus sinensis* quality-related molecular changes during consecutive developmental periods, including young fruit, fruit-coloring onset and fruit delayed-harvest for two months, during which fruit remained on the trees.

**Results:**

The time-course analysis for the fruit developmental periods indicated a complex, dynamic gene expression pattern, with the numbers of differentially expressed genes (DEGs) between the two cultivars being 119, 426 and 904 at the three continuous stages tested during fruit development and ripening. The continuous increase in total soluble solids over the course of fruit development was correlated with up-regulated *sucrose phosphate synthase* (SPS) transcription levels in both cultivars. Eleven differentially expressed genes between the two cultivars involved in the flavonoid pathway were significantly enriched at the onset of the fruit-coloring stage when anthocyanins were detected in blood orange alone. Among 5185 proteins, 65 up-regulated and 29 down-regulated proteins were co-expressed with their cognate mRNAs with significant transcription and protein expression levels when the fruits from the two cultivars were compared at the fruit delayed-harvest stage. Additionally, important genes participating in the γ-aminobutyric acid (GABA) shunt were activated in blood orange at two significant expression levels in the fruit delayed-harvest stage. Thus, organic acids in fruit continuously decreased during this stage.

**Conclusions:**

This research was the first to provide a more comprehensive understanding of the differentially expressed genes involved in anthocyanin, sucrose and citrate metabolism at the transcriptome and proteome levels in *C. sinensis*, especially during the fruit delayed-harvest stage.

**Electronic supplementary material:**

The online version of this article (10.1186/s12864-017-4366-2) contains supplementary material, which is available to authorized users.

## Background

Among fruit tree species, *Citrus* is the most important subtropical fruit tree worldwide. Most *Citrus* species are apomictic, polyembryonic and propagated vegetatively. *Citrus* cultivars are primarily derived from bud sports, which are caused by spontaneous or induced somatic mutations. As the largest transposable elements in plant genomes, retrotransposons are thought to be involved in *Citrus*’ genetic instability and genome evolution, especially in *Citrus sinensis*, which is prone to bud mutations [1]. Blood orange originated from a retrotransposon insertion that promotes *ruby* gene expression in *C. sinensis*; *ruby* encodes a MYB-type transcription factor that activates anthocyanin production [2]. Almost all of the natural variation in pigmentation caused by anthocyanins in *Citrus* species can be explained by differences in the activity of the *ruby* caused by point mutations, deletions and insertions resulting from transposable elements [3]. Anthocyanin production can also be transcriptionally regulated by the MYB-bHLH-WD40 (MBW) transcription factor complex in blood orange, similar to other fruit trees rich in anthocyanins [4].

Anthocyanins, a type of flavonoid, contain an abundance of functional phytochemicals and occur in many fruit trees. Anthocyanins have a higher antioxidant capacity against oxidative stress induced by excess reactive oxygen species, such as superoxide radicals and hydrogen peroxide; thus, the human body might be protected from oxidative injury by consuming fruits rich in anthocyanin. Previous studies performed by HPLC with spectrophotometric detection showed that cyanidin 3-glucoside is predominant followed by minor components in blood orange juice [5]. Blood orange juice contains anthocyanins in differing concentrations depending on the variety, environment and cultivation practices. During post-harvest cold storage, dihydroflavonol channeling towards anthocyanin production in blood orange is boosted, providing more leucoanthocyanidins to enzymes downstream in the pathway [6]. However, the genes that participate in anthocyanin production and regulation were unclear during *C. sinensis* fruit delayed-harvest from on-tree storage.

Fruit delayed-harvest practices for different *Citrus* species are widely used in China to prolong harvesting seasons and improve fruit quality. However, the important genes involved in fruit-quality improvements during fruit delayed-harvest are poorly understood. Thus, comprehensive studies on the genes and proteins participating in the continuous development from young fruit to delayed-harvest fruit are necessary to fully understand the biological processes associated with delayed-harvest practices. Using an RNA sequencing (RNA-seq) technology, an orange-pericarp pomelo revealed new insights into the molecular regulation of β-carotene accumulation in the rind [7]. Moreover, only two up-regulated differentially expressed proteins, chalcone synthase and flavanone 3-hydroxylase, were found in blood orange through a proteomics analysis using a combination of 2-DE and LC-MS/MS approaches [8]. More recently, an LC-MS/MS-based shot-gun proteomics approach was used to quantify differential protein synthesis during *Citrus* fruit development based on the iCitrus database for the high-throughput identification of *Citrus* proteins [9]. However, even though this database was created by merging expressed sequence tags (ESTs), available unigenes and protein sequences, it was only able to identify 1394 unique citrus proteins expressed in fruit juice sac cells; this finding is observed because the iCitrus database is not complete and is limited to sequences isolated from specific libraries [10]. Thus, it is necessary to combine proteomics and transcriptomics technologies to perform a more complete and reliable characterization of the biological systems in *Citrus* fruit than previous studies when the genome sequencing of sweet orange was achieved. The objective of this work was to better understand the molecular changes that occur during fruit development and ripening based on transcriptome and proteome approaches using developmental stage to stage and specific cultivar to cultivar comparisons.

## Results

### Fruit quality measurements during fruit ripening

**Fig. 1:**
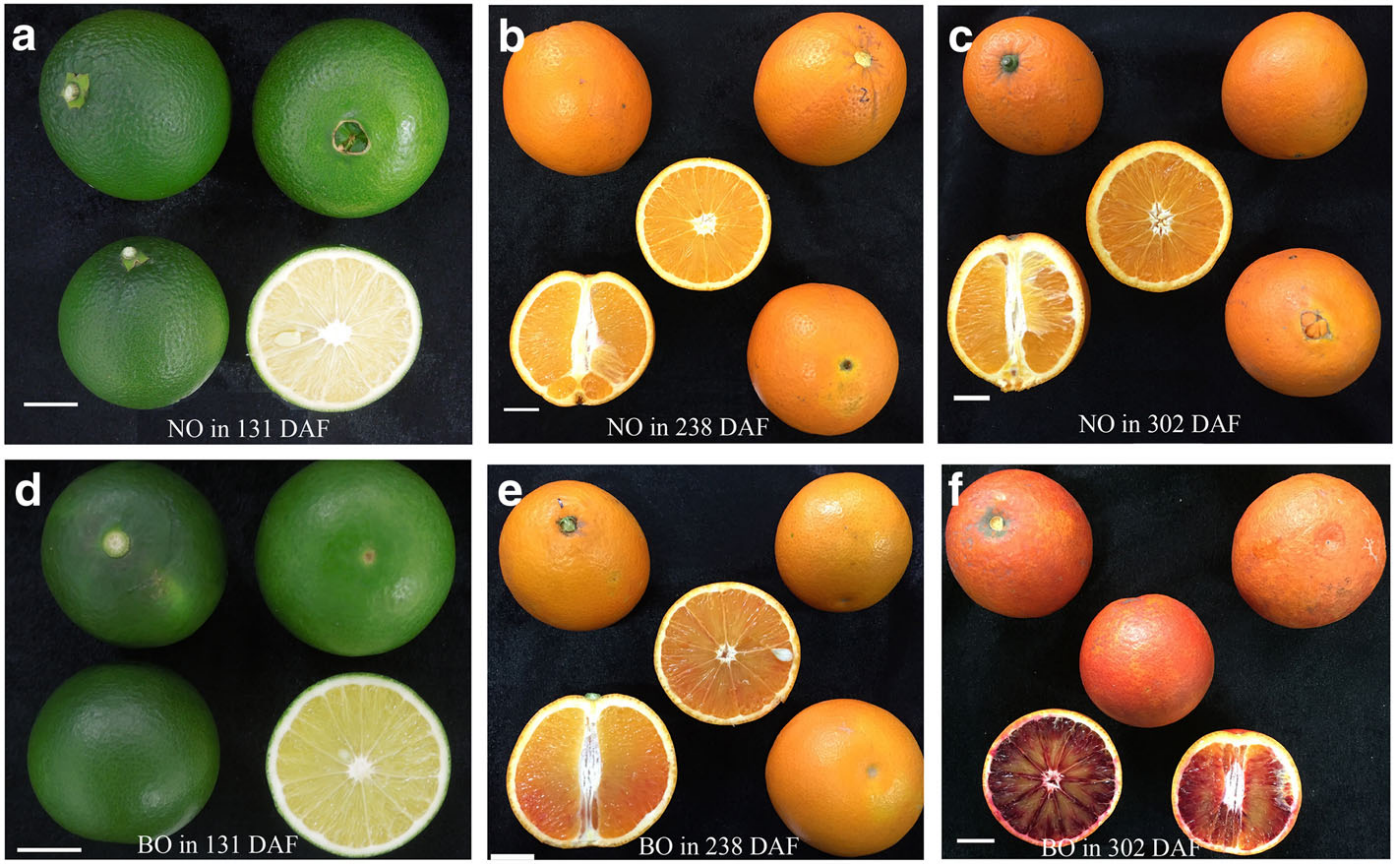
Fruits from two cultivars harvested during fruit development and ripening, respectively **a** young blond orange fruits at 131DAF **b** mature blond orange fruits at 238DAF **c** blond orange fruits delayed-harvest at 302 DAF **d** young blood orange fruits at 131DAF **e** onset of blood orange fruit-coloring at 238 DAF **f** blood orange fruits delayed-harvest at 302 DAF. White bar = 2 cm

‘Zaohong’ (blood orange) and ‘twenty-first century’ (common blond orange) were collected from our experimental orchard at 131 DAF, 238 DAF and 302 DAF (Fig. 1). In this study, the ratios of the total soluble solids (TSS) to the titratable acid (TA) were determined to evaluate fruit maturity. The blond orange had a significantly higher TSS/TA ratio throughout fruit development and ripening than ratio in blood orange (Additional file 1). At the young fruit stage (131 DAF), the flesh of the two cultivars had similar light yellow colors. Beginning at 238 DAF, the flesh of blood orange underwent fruit pigmentation. At this moment, the blond orange became fully ripe (TSS/TA = 13.97), while the blood orange was close to full maturity (TSS/TA = 9.29). After two months of fruit delayed-harvest, the TSS/TA ratio in the blood orange rapidly increased (TSS/TA = 12.87), whereas it was still lower than that of the blond orange (TSS/TA = 14.56). Eventually, at 302 DAF, darker purple-colored rind and flesh formed in the blood orange, while the flesh in the blond orange became darker orange in color.

### RNA-seq results

In total, 132.83 GB of clean data was obtained from the 18 cDNA libraries. On average, approximately 6.12 GB of clean data per library (Q30 > 85.68%) was generated. Thus, from 41,055,766 to 74,325,552 reads per library were produced, out of which 68.01 to 76.02% of the reads were uniquely mapped to the reference genome (Additional file 2). Three biological replicates were collected for each cultivar during each fruit development and ripening period. Pearson’s correlation coefficients among the three biological replicates were high (γ = 0.84–0.99), indicating that the sequencing quality allowed for further analysis (Additional file 3).

### DEGs obtained by comparing the two cultivars

**Fig. 2:**
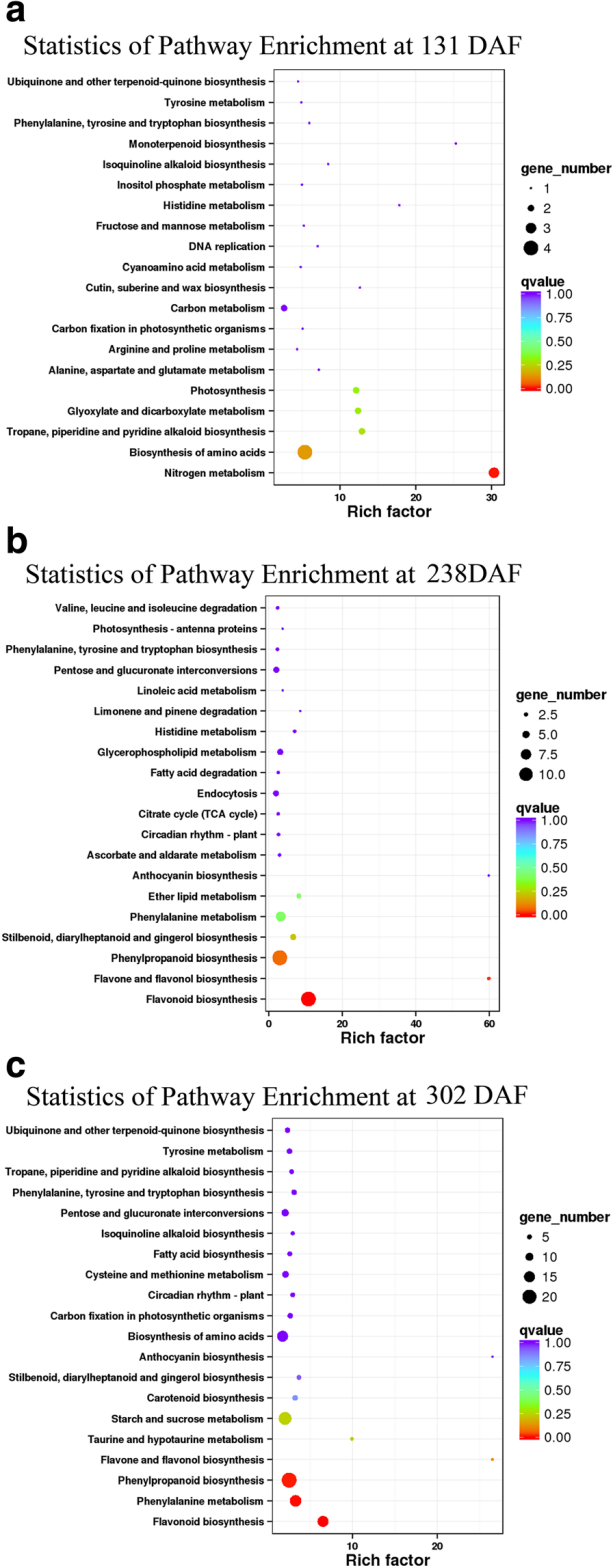
DEGs involving into KEGG pathway enrichment analysis in comparison with two cultivars along fruit development and ripening. **a** DEGs participating in nitrogen metabolism pathway significantly enriched at 131 DAF. **b** DEGs participating in flavonoid pathway significantly enriched at 238 DAF. **c** DEGs participating in phenylpropanoid–flavonoid pathway significantly enriched at 302 DAF

When comparing blood orange to blond orange, 119 (24 up-regulated and 95 down-regulated genes), 426 (225 up-regulated and 201 down-regulated genes) and 904 (558 up-regulated and 346 down-regulated genes) differentially expressed genes (DEGs) were found at 131 DAF, 238 DAF and 302 DAF, respectively (Additional file 3). The young fruit from the two varieties had similar physiological conditions prior to fruit maturation; therefore, only three DEGs participating in the nitrogen metabolism pathway were significantly enriched (Q value <0.05, Fig. 2a). Subsequently, 11 DEGs involved in the flavonoid pathway (KEGG) were significantly enriched at the fruit-coloring onset stage (Q value <0.05, Fig. 2b). The anthocyanin contents in blood orange were detectable at this stage. Finally, 21, 6 and 15 DEGs involved in the phenylpropanoid, phenylalanine and flavonoid biosynthesis pathways, respectively, were significantly enriched during the fruit delayed-harvest stage (Q value <0.05, Fig. 2c). As a result, greater anthocyanin contents were observed in blood orange at 302 DAF, as more DEGs are involved in the phenylpropanoid–flavonoid pathway using the precursor phenylalanine. However, when comparing the two cultivars, other DEGs that participate in the sucrose and citrate metabolic pathways were not significantly enriched as the fruits ripened. This finding may be the result of the limited genetic variability between these two *C sinensis* cultivars, as a transposon insertion alone causes the pigmentation mutation in blood orange.

**Fig. 3:**
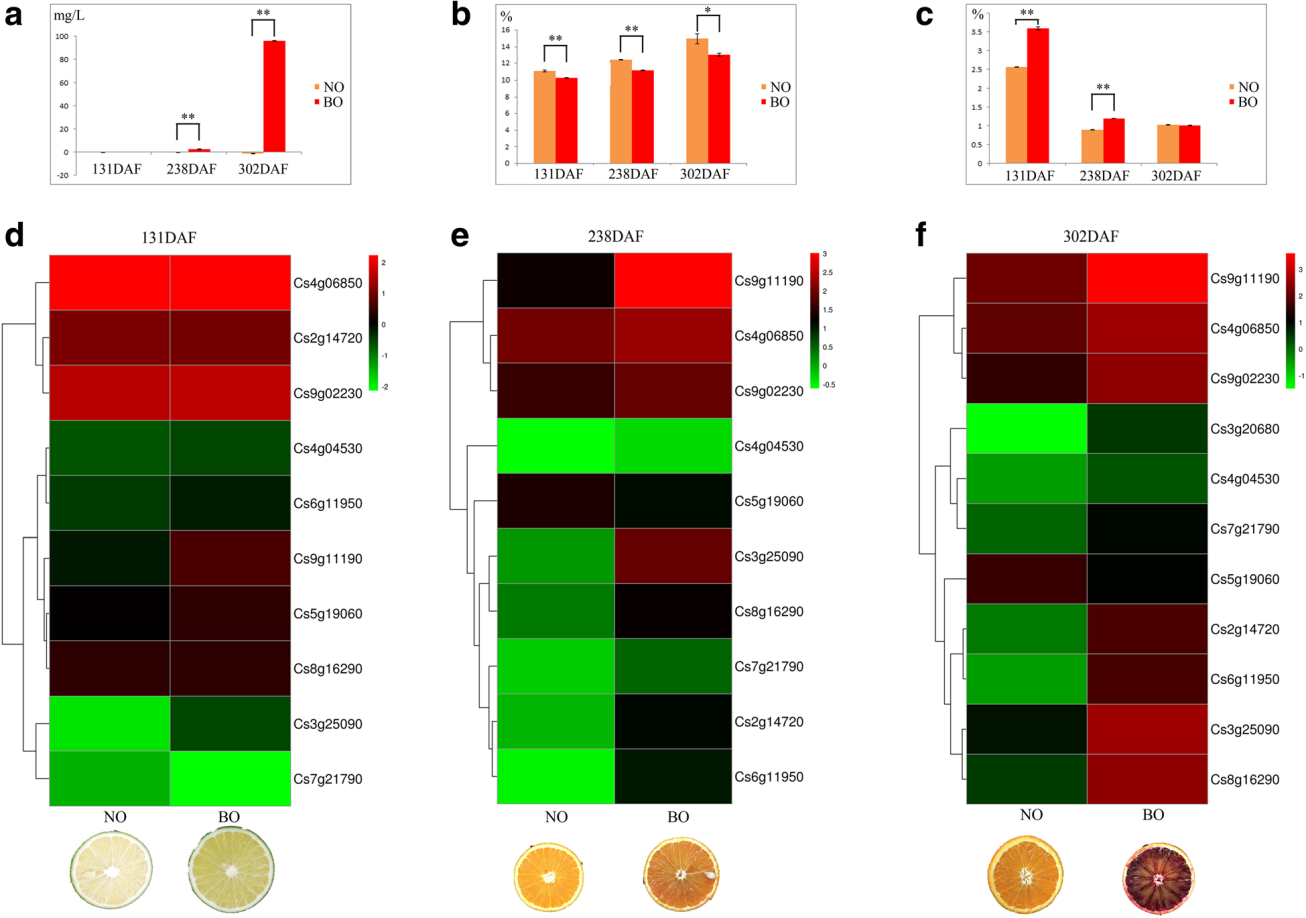
Fruit quality measurements and hierarchical clustering of DEGs in comparison with two cultivars during fruit development and ripening. **a** Measurement of anthocyanin contents **b** Measurement of total soluble solids **c** Measurements of titratable acids **d** Hierarchical clustering analysis of DEGs between two cultivars at 131 DAF **e** Hierarchical clustering analysis of DEGs at 238 DAF **f** Hierarchical clustering analysis of DEGs at 328 DAF. BO represented by blood orange NO represented by navel orange

The anthocyanin contents in both varieties during the young fruit stage (131 DAF) were undetectable (Fig. 3a). Afterwards, anthocyanin was only found in the blood orange, not in blond orange. At the onset of the fruit-coloring stage (238 DAF), anthocyanin levels were 2.28 mg/L and later increased rapidly by 95.85 mg/L after two months of fruit delayed-harvest (302 DAF, Fig. 2a). As a result, eights DEGs, including *phenylalanine ammonia-lyase* (*PAL*, Cs8g16290, Cs6g11950), *cinnamate 4-hydroxylase* (Cs4g04530), *4-coumarate:CoA ligase* (*4CL*, Cs7g21790), *chalcone synthase* (*CHS*, Cs3g20680, Cs9g11190, Cs2g14720) and *dihydroflavonol 4-reductase* (*DFR*, Cs3g25090), had significantly higher fold changes in blood orange at 238 DAF, which was in agreement with detectable anthocyanin levels (Fig. 3f). However, the above DEGs were not significantly different during the young fruit stage (131 DAF).

**Fig. 4:**
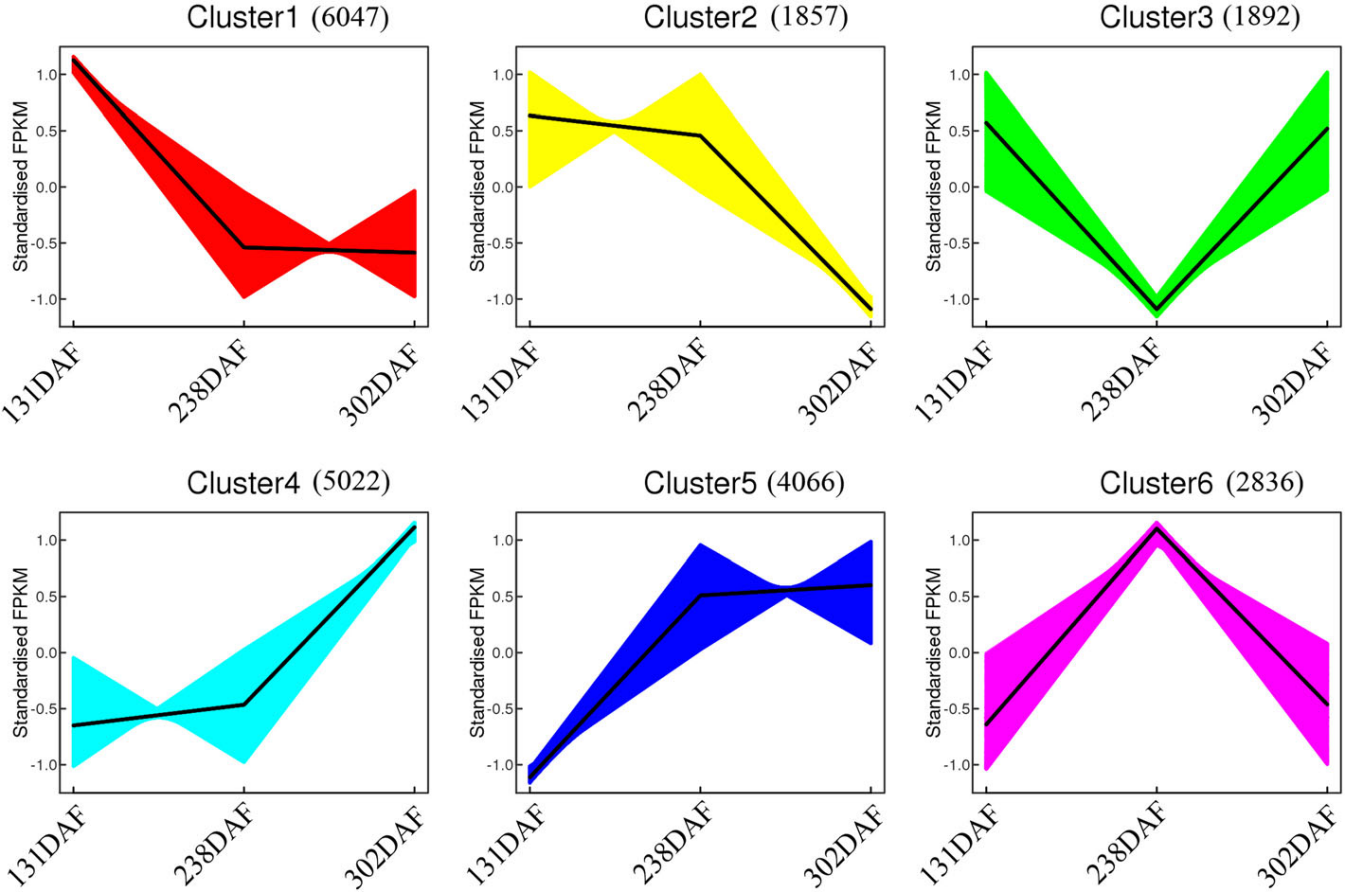
Clustering analysis of the gene expression patterns for blood orange in comparison with fruit developmental stage to stage (The clustering numbers of genes in each parenthesis)

The TSS was continuously elevated in both cultivars during fruit development and ripening. Interestingly, the greatest TSS levels occurred in the two cultivars during the fruit delayed-harvest stage (Fig. 3b). When comparing the two cultivars, *sucrose phosphate synthase* (*SPS*, Cs5g19060) transcription levels were higher in blond orange at 238 DAF (Fig. 3b). However, TA persistently decreased from 3.59% to 1.01% in blood orange and 2.56% to 1.02% in blond orange during fruit development (Fig. 3c). *Citrate synthase* (*CS*, Cs9g02230) transcription levels displayed a significantly higher fold change in blood orange at 238 DAF, which coincided with the higher TA levels in blood orange (Fig. 4f). Highest TA levels were observed in blood orange during the young fruit stage. However, by the delayed-harvest stage, there were no significant differences between the two cultivars, partially because the citrate degradation pathway was activated in blood orange, which accompanies fruit development and causes decreased acidity.

### Comparison of blood orange DEGs during stage to stage development

By comparing fruit development stage to stage, the gene ontology classifications for the DEGs in blood orange at the fruit coloring-onset stage (238 DAF vs. 131 DAF) and at the fruit delayed-harvest stage (302 DAF vs. 238 DAF) were involved in biological processes, molecular functions and cellular components (Additional file 4). In particular, 2372 DEGs were identified at the fruit coloring-onset stage, though only 690 DEGs were found at the delayed-harvest stage. Of these DEGs, 38 related to sucrose metabolism were also classified into biological process at the fruit-coloring onset stage, including down-regulated Cs3g22270 (*alkaline/neutral invertase*) and Cs4g06630 (*pectinesterase*) and up-regulated Cs5g19060 (*sucrose-phosphate synthase*), Cs5g33470 (*sucrose synthase*) and Cs7g05690 (*sucrose-phosphate synthase*). Eight DEGs related to citrate metabolism were also classified into biological process at this stage, including up-regulated Cs1g03610 and Cs8g03230 (*malate dehydrogenase*) as well as Cs9g02230 (*citrate synthase*) and down-regulated Cs2g03260 (*pyruvate dehydrogenase*). Furthermore, 14 DEGs related to sucrose metabolism were classified into biological process at the fruit delayed-harvest stage, including up-regulated Cs5g33420 (*pectinesterase*) and Cs4g06850 (*sucrose synthase*). However, only up-regulated Cs1g20920 (*phosphoenolpyruvate carboxykinase*), which is involved in the gluconeogenesis pathway, was shown to produce sucrose in blood orange at this stage.

The expression patterns of 21,720 and 21,492 transcripts identified in blood orange and blond orange, respectively, were determined using the K-means clustering method during fruit development and ripening (Fig. 4). Six different clusters of expression patterns were recognized in both cultivars. The clustering showed recognizable patterns for decreasing or increasing expression as fruit developed. Clusters 1 and 2 in blood orange were clearly decreasing (7904 genes), while Clusters 4 and 5 were increasing (9088 genes). However, Clusters 3 and 6 had irregular expression patterns (Fig. 4). Eight anthocyanin biosynthesis-related genes were observed in Cluster 4 during blood orange fruit development; this finding clearly indicated that their transcription levels were more rapidly up-regulated in fruit during the delayed-harvest stage than the previous development stage, in which more anthocyanin was accumulated. In contrast, the expression pattern for Cs5g19060 (*SPS*), which is involved in sucrose biosynthesis, was more quickly increased during the fruit coloring-onset stage than in the later development stage (Cluster 5). Cs9g02230 (*CS*) and orange1.1 t01622 (*GAD*, *Glutamate decarboxylase*) were also found in Cluster 4, which indicates that the two genes play more important roles in citrate production and degradation during the delayed-harvest period. Moreover, Cs5g19060 had the same expression pattern in blond orange as in blood orange (data not shown). In contrast, Cs9g02230, which is involved in citrate production, showed decreased expression during blond orange fruit development (data not shown).

### Confirmation of fruit quality-related genes in blood orange by qPCR

**Fig. 5:**
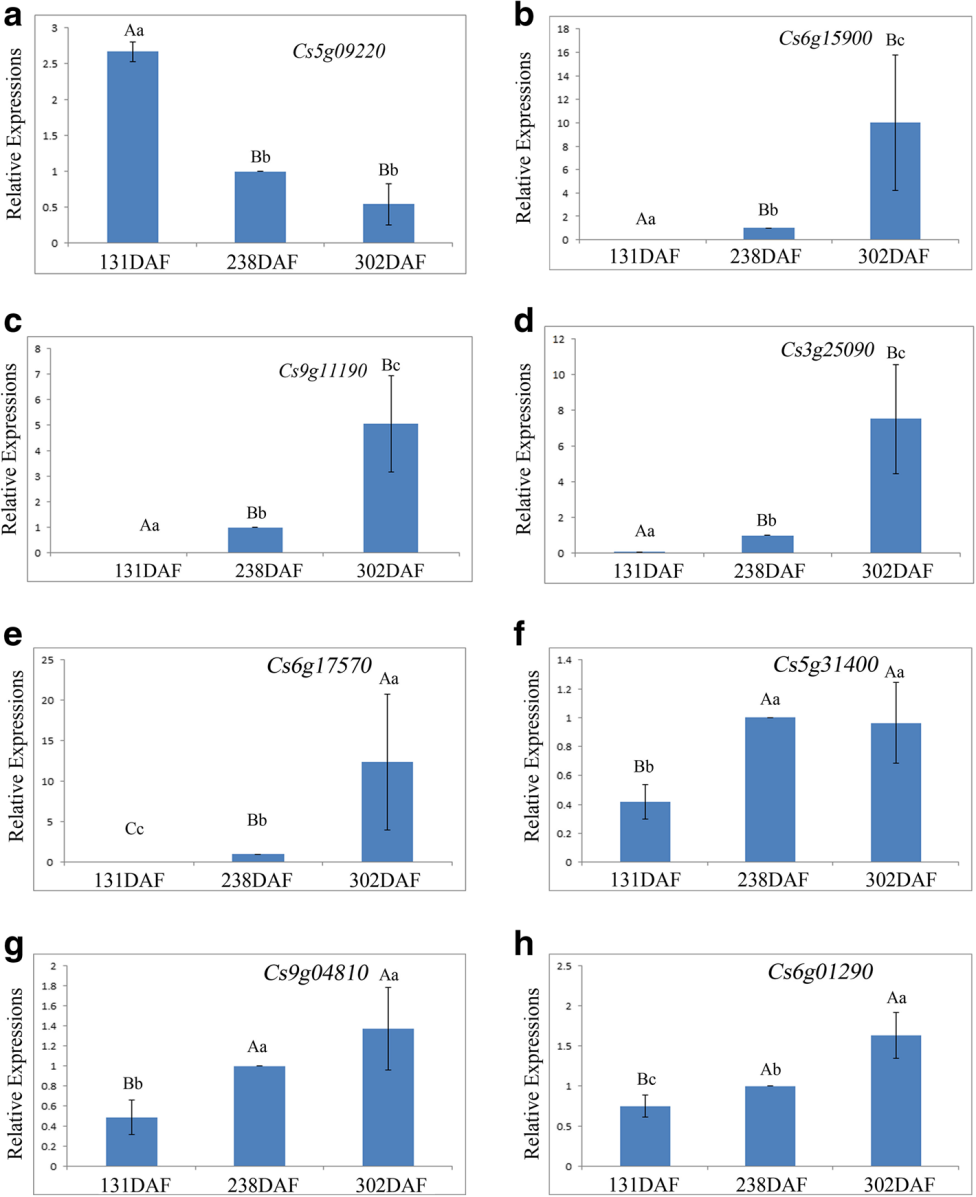
The relative expression folds of each gene determined by qPCR in blood orange along the course of fruit development. **a** Cs5g09220, acid invertase, relative expression folds during fruit development, **b** Cs6g15900, glutathione S-transferase, relative expression folds during fruit development, **c** Cs9g11190, chalcone synthase, relative expression folds during fruit development, **d** Cs3g25090, dihydroflavonol 4-reductase, relative expression folds during fruit development, **e** Cs6g17570, MYB type transcription factor, relative expression folds during fruit development, **f** Cs5g31400, bHLH type transcription factor, relative expression folds during fruit development, **g** Cs9g04810, WD40 repeat transcription factor, relative expression folds during fruit development, **h** Cs6g01290, ATP-citrate synthase, relative expression folds during fruit development

The fold changes (log_2_ FC) of eight genes in blood orange during the fruit-coloring onset stage (238 DAF vs. 131 DAF) and fruit delayed-harvest stage (302 DAF vs. 238 DAF) were determined using the FPKM values obtained from fruit transcriptome sequencing (Table 1). The fold changes for Cs5g09220 (*acid invertase*), Cs5g31400 (*bHLH type transcription factor*) and Cs9g04810 (*WD40 repeat transcription factor*) in blood orange were significantly changed at 238 DAF but not at 302 DAF (Table 1), which agreed with the qPCR results (Fig. 5). In addition, the fold changes in Cs3g25090 (*dihydroflavonol 4-reductase*), Cs9g11190 (*chalcone synthase*) and Cs6g15900 (*glutathione S-transferase*) in blood orange increased significantly at both 238 DAF and 302 DAF (Table 1), which also agreed with the qPCR results (Fig. 5). The transcriptional changes in Cs5g09220 (*acid invertase*) determined by qPCR were significantly negatively correlated with the TSS changes (γ = −0.838, *P* = 0.05) and Cs6g01290 (*ATP-citrate synthase*) was also negatively correlated with TA changes throughout fruit development (γ = −0.705, *P* = 0.034). Furthermore, the relative expression levels of anthocyanin production-related enzymatic genes, including Cs3g25090, Cs9g11190 and Cs3g25090, were significantly correlated with anthocyanin accumulation during fruit ripening (Table 2). In comparison, only two out of three regulator genes (Cs6g17570 and Cs5g31400) showed significant correlations with anthocyanin production (Table 2). Thus, FPKM value of the each transcript determined by RNA-seq was reliable for quantitatively studying DEG dynamic changing.

**Table 1:** Fold changes of DEGs identified by RNA-seq in blood orange during fruit development and ripening

Gene ID	Annotation	FDR	log2 FC	DAF	Changes at transcription level by qPCR
Cs5g09220	Acid invertase	3.29E-04	−1.935	238	down^**^
Cs6g15900	Glutathione S-transferase	1.71E-67	6.313	238	up^**^
Cs9g11190	Chalcone synthase	2.71E-136	7.62	238	up^**^
Cs3g25090	Dihydroflavonol 4-reductase	1.93E-59	8.18	238	up^**^
Cs6g17570	R2R3 MYB transcription factor	2.83E-05	4.115	238	up^**^
Cs5g31400	bHLH type transcription factor	0.000077719	0.995	238	up^**^
Cs9g04810	WD repeat transcription factor	0.000365	0.849	238	up^**^
Cs6g01290	ATP-citrate synthase	0.00000291	0.786	238	up^**^
Cs5g09220	Acid invertase	either > 0.05	or < 0.75	302	down
Cs6g15900	Glutathione S-transferase	0.0034835278	2.498	302	up^*^
Cs9g11190	Chalcone synthase	0.0015239598	1.87	302	up^*^
Cs3g25090	Dihydroflavonol 4-reductase	3.26E-04	2.304	302	up^*^
Cs6g17570	R2R3 MYB transcription factor	either > 0.05	or < 0.75	302	up^**^
Cs5g31400	bHLH type transcription factor	either > 0.05	or < 0.75	302	up
Cs9g04810	WD repeat transcription factor	either > 0.05	or < 0.75	302	up
Cs6g01290	ATP-citrate synthase	either > 0.05	or < 0.75	302	up^*^

Asterisks represents statistically significant differences (^*^ *P* < 0.05; ^**^ *P* < 0.01)

**Table 2:** Pearson’s correlation analysis between the transcription levels of genes with anthocyanin contents

	DFR	CHS	GST	R2R3 MYB TF	bHLH type TF	WD40 repeat TF
Anthocyanin Pearson’s correlation	0.915^**^	0.914^**^	0.855^**^	0.798^**^	0.459	0.708^*^
Significant (two-tail test)	0.001	0.003	0.001	0.01	0.214	0.033

Asterisks represents statistically significant differences (^*^ *P* < 0.05; ^**^ *P* < 0.01)

### Important gene cross-talk between the protein and transcription levels

Using proteomics profiling approach, expression ratios from a cultivar to cultivar comparison during the fruit delayed-harvest stage (302 DAF) identified 5185 proteins, and 4648 proteins were quantified. Pearson’s correlation coefficients for protein expression among the three biological replicates for each sample were considerably high (γ = 0.56–0.75). We also performed a direct comparison of the transcriptome and proteome data obtained during the fruit delayed-harvest stage. Concordant tests revealed a correlation between 4248 pairs of proteins and cognate mRNAs (γ = 0.6833, *P* < 0.01, Additional file 5). However, the Pearson’s correlation was even higher (γ = 0.886, *P* < 0.01) when 208 pairs of co-expressed proteins and their cognate mRNAs were compared (concordant dots in red color from 3 and 7 plots), regardless of the direction of the change (up-regulated or down-regulated) or the statistical significance of the differential expression levels, in which genes were normally not effected by post-transcriptional regulation and post-translational modification (Additional file 5). Consequently, the transcriptome and proteome data were reliable enough for the combined analysis. The expression ratios for proteins above 1.2 or below 0.8333 (*P* < 0.05) between the two cultivars were considered to be differentially expressed. Furthermore, transcript fold changes above 2 (P < 0.01) found in the comparison between the two cultivars were also assigned as DEGs. Thus, 857 differentially expressed proteins were identified, out of which 469 proteins were up-regulated and 388 proteins were down-regulated. Simultaneously, 904 DEGs were found, out of which 558 transcripts were up-regulated and 346 transcripts were down-regulated. Among the 5185 proteins, 65 proteins and their cognate mRNAs were up-regulated, while 29 proteins and their cognate mRNAs were down-regulated at significant differential expression levels between the two cultivars during the fruit delayed-harvest stage (Additional file 6).

**Fig. 6:**
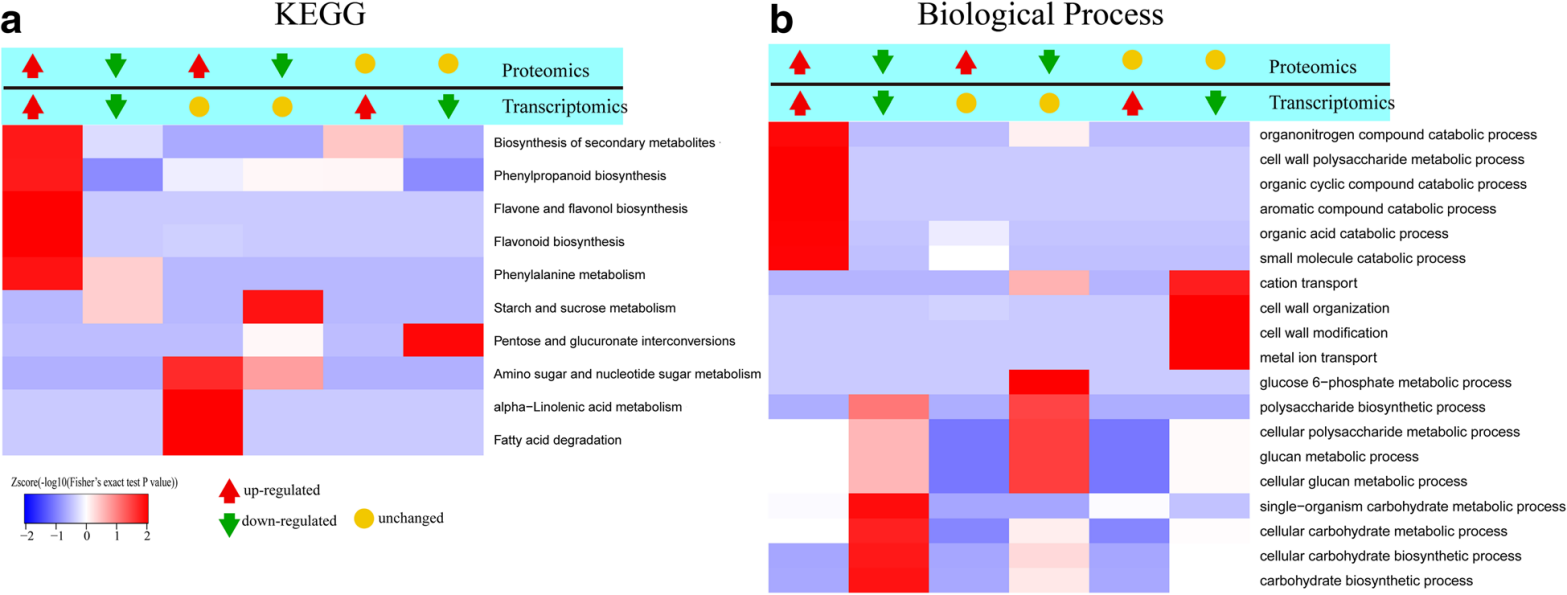
Significant enrichment analysis of DEGs for each pair of protein and cognate mRNA based on the direction of the change. **a** DEGs involving into different KEGG pathway enriched significantly. **b** DEGs classified into different biological process enriched significantly

The relative expression folds for each mRNA and their cognate protein obtained from transcriptomic and proteomic combined analysis shown the co-expression patterns of DEGs participating in the important biological pathway. Cs3g25090 (*DFR*), Cs1g25280 (*F3H*, *flavanone 3-hydroxylase*), Cs5g09970 (*LDOX*, *leucoanthocyanidin dioxygenase*), Cs2g29510 (*F3’5’H*, *flavonoid 3′,5′-hydroxylase*), Cs9g11190 (*CHS*) and Cs5g11730 (*F3H*, *flavanone 3-hydroxylase*) had up-regulated co-expression of their proteins and their cognate mRNAs that were enriched in the flavonoid biosynthesis pathway when comparing blood orange to navel orange (Fig. 6a). Moreover, the down-regulated co-expression of Cs5g19060 (*SPS*) at the RNA and protein expression level was enriched in the carbohydrate biosynthetic process from biological process (Fig. 6b), while decreased *SPS* expression was correlated with a lower TSS level in blood orange compared to navel orange. Besides, the up-regulated co-expression of Cs9g02230 (*CS*) and orange1.1 t01622.1 (*GDA*) at two significant expression levels was enriched in the organic acid catabolic process when comparing the two cultivars (Fig. 6b).

## Discussion

From young fruit to delayed-harvest fruit, the blood orange’s pulp underwent a rapid change in color, changing from yellow to dark purple, due to the degradation of chlorophyll and the biosynthesis of anthocyanins, primarily cyanidin-3-glucoside. Previous studies indicated that fruit anthocyanin contents at full maturity were highly correlated with the expression of anthocyanin biosynthesis genes in many fruit trees, including peach [11], grape [12], apple [13], litchi [14] and Chinese bayberry [15]. When blood orange fruits were stored at 4 °C for 77 d, transcriptome modifications were induced to enhance the phenylpropanoid pathway, including those reactions involved in the anthocyanin biosynthesis and phenylalanine pathways that feed it [16]. Glutathione S-transferases are involved in the vacuolar import of anthocyanins and are active against cyanidin-3-O-glucoside [17]. Enzymatic genes (*PAL*, *CHS*, *DFR*, *ANS* and *UFGT*) participate in anthocyanin biosynthesis in blood orange, which is a cold-regulated pathway [18]. In this study, the anthocyanin contents in fruit (delayed-harvest for two months) rapidly increased, while the orchard’s environmental temperature decreased by 6–7 °C. Seven co-expressed proteins and their cognate mRNAs (*PAL*, *CHS*, *F3’H*, *F3’5’H*, *DFR*, *ANS* and *UFGT*) were up-regulated, with high correlations between the transcription and protein expression levels (Additional file 7). The flavonoid biosynthesis pathway in *Arabidopsis* involves a plethora of functionally well-known catalytic and regulatory proteins, some of which participate in the flavonoid pathway by directly interacting with MBW complex components [19]. In *Arabidopsis*, TCP3 forms a heterodimer with MYBL2; it is also possible that TCP3 reinforces the formation of the MBW complex by binding to MYBL2, thereby freeing bHLHs to produce anthocyanin [20]. In the present study, R2R3 MYB, bHLH type and WD40 repeat transcription factors had higher transcription levels in blood orange while fruit ripened; these transcription factors may form a regulatory complex to promote downstream anthocyanin production-related enzymatic gene expression. Throughout blood orange fruit development, catalytic and modulator genes were up-regulated in response to lower orchard temperatures and promoted increased anthocyanin production, particularly during the fruit delayed-harvest stage.

Similar to post-harvest fruit treated in cold storage (detached from the tree) [21], the increasing TSS changes in the two cultivars were also observed during the fruit delayed-harvest stage (when fruits still attached on the tree). In another study, organic acid and amino acid accumulation shifted towards sugar synthesis during the mature stage in navel orange ‘Washington’ fruit development [22]. Besides, the activity of *SPS* in *Citrus unshiu* paralleled the transcription levels of *CitSPS1* and *CitSPS2*, suggesting that the expression of these two genes might have important roles in determining sucrose composition or accumulation [23]. In the present study, *SPS* (*Cs5g19060*) was significantly up-regulated at the fruit-coloring stage (238 DAF) but did not significantly change at the fruit delayed-harvest stage (302 DAF) in either cultivar based on the FPKM data. Simultaneously, the continuous increase in the TSS contents during blond orange ripening was significantly correlated with *SPS* expression changes based on the FPKM data (γ = 0.915, *P* = 0.001), though it had a lower correlation in blood orange (γ = 0.559, *P* = 0.118) during fruit ripening. Thus, *Cs5g19060* might play an important role in sucrose metabolism in *C. sinensis*, especially in navel *oranges.*

Once aconitase enzymatic activity is inhibited in *Citrus* fruit, excess citrate is converted into amino acids through the induction of the GABA shunt, further supporting previous reports on the fate of citrate during the acid decline stage [24]. In the GABA shunt, *GAD* participates in regulating cytosolic pH during fruit ripening. Orange1.1 t04401 (*GS*, *Glutamine-dependent NAD*(+) *synthetase*) was up-regulated in both cultivars to produce glutamine using citrate as the precursor during fruit development and ripening. Additionally, the GABA shunt was activated by Cs2g12320 (*pyruvate kinase*), Cs9g02230 (*CS*) and orange1.1 t01622.1 (*GAD*) in blood orange at the fruit delayed-harvest stage at both the transcriptional and protein expression level to biosynthesize γ-aminobutyric acid through a proton-consuming reaction (Additional file 8). Microarray and real time-PCR data indicated that the expression levels of genes encoding *glutamate decarboxylase* and *GABA aminotransferase* were the highest during the acid loss period, which is consistent with the GABA shunt being more active or mostly functioning during *Citrus clementine* fruit ripening [25]. Consequently, organic acid levels in the two cultivars gradually decreased following sweet orange development after two months of delayed-harvest.

Abscisic acid is an important regulator of the onset of *C. sinensis* fruit degreening and carotenoid biosynthesis [26]. In the present study, *9-cis-epoxycarotenoid dioxygenase* (Cs2g03270, Cs5g14370 and Cs7g01700), which is involved in abscisic acid biosynthesis, had a continuously down-regulated expression pattern in both cultivars as the fruits developed. Nevertheless, Cs2g03270 displayed lower expression levels (at both transcriptional and protein levels) during late ripening in blood orange than in navel orange at the fruit delayed-harvest stage. Ethylene is required for the onset of anthocyanin accumulation, fruit swelling, and decreased acidity that is associated with the ripening of grape berries [27]. In *C. sinensis*, the transition from the ethylene biosynthesis system II-like behaviors for young fruitlets to system I behaviors in mature and detached fruits appears to be under developmental control [28]. *1-Aminocyclopropane-1-carboxylate oxidase* (*ACO*), which participates in ethylene biosynthesis, was down-regulated in a late-ripening sweet orange cultivar compared with its parent [29]. However, the transcription and protein expression levels of *ACO* (Cs2g20590) were higher in the late-ripening blood orange during the delayed-harvest stage. Moreover, the expression pattern for *ACO* (Cs4g13870) in both cultivars more rapidly increased at the fruit-coloring onset stage than during later developmental stages, which suggests that ethylene also plays an important role in the ripening process in non-climacteric fruit. Following fruit development and ripening, *C. sinensis* also modulated the expression levels of genes involved in cell wall modification. In the two *C. sinensis* cultivars, the *pectinesterase* (Cs4g06630) gene was highly expressed prior to full fruit maturation, and was continuously down-regulated from the fruit-coloring to delayed-harvest stage, which is similar to its expression pattern in tomato [30]. Two genes encoding *xyloglucan endotransglycosylase* (Cs4g03200 and Cs8g03550) displayed up-regulated expression patterns during fruit development and ripening, indicating their probable roles in cell wall degradation during ripening in both cultivars, as well as in other sweet orange cultivars [31]. Furthermore, the gene encoding *endo-1,4-beta-glucanase* (Cs6g19070) was up-regulated in both cultivars over the course of fruit development and ripening.

With the data provided by the comprehensive analysis using transcriptomic and proteomic profiling, important genes involved in the development and regulation of these fruit-quality attributes were characterized, which can further contribute to understanding *C. sinensis* biology. In total, 59 mRNAs/proteins in fruit ripening significantly changed at one level, either the transcript or protein level, indicating a poor correlation between transcripts and proteins in *C. sinensis* [29]. In this study, 904 genes from a comparison between the two cultivars had significantly differential expression at the transcription level, but only 94 differentially expressed proteins were co-expressed with their cognate mRNAs. Therefore, it is likely that other factors, such as the regulation of mRNA translation and post-translational processing, have a more relevant role than the time-lag between transcription and translation on the weak correlation between the transcriptome and proteome data [32].

## Conclusions

This study presented a dynamic picture of *C. sinensis* via transcriptome analysis using developmental stage to stage and cultivar to cultivar comparisons. The co-expressed proteins and cognate mRNAs were further identified using transcriptomic and proteomic technologies, especially at the fruit delayed-harvest on-tree storage stage. The important genes, which were involved in anthocyanin biosynthesis, sucrose production, citrate degradation, signal transduction and cell wall metabolism, were differentially expressed at both the transcriptomic and proteomic levels after two months of the fruit delayed-harvest to promote better fruit taste. This report describes the first time that integrated analysis combining transcriptome and proteome profiling has been used to comprehensively understand important molecular events that are relevant to fruit-quality attributes in sweet orange during fruit development and ripening.

## Methods

### Plant materials and fruit quality measurements

*Citrus sinensis* cv. Zaohong, a blood orange cultivar selected from a ‘Tarocco’ nucellar line and *C. sinensis* cv. twenty-first century, a blond orange selected from a bud sport from a ‘Washington’ navel orange, were the two cultivars obtained by our breeding program. The fruits were collected from 7-year-old *‘*Zaohong’ grafted onto *Citrus junos* at 131 days after flowering (DAF; stage I = August 5th, 2016), 238 DAF (stage II = December 4th, 2016) and 302 DAF (stage III = February 3rd, 2017) at a Xindu experimental orchard (Sichuan Province, China). Fruit were simultaneously collected from 10-year-old *‘*twenty-first century’ grafted onto *C. junos* in the same orchard at the same developmental stages. Both ‘Zaohong’ blood orange and ‘twenty-first century’ navel orange are normally fully ripe at the beginning of December in Sichuan Province. The total titratable acid, total soluble solid and anthocyanin contents in the fruit juice were measured based on a previous protocol [4].

### Library preparation and sequencing

An aliquot (6 μg) of total RNA from each cultivar, with three independent replicates, was extracted using an RNA isolation kit (Huayueyang, Beijing, China). A total of 1 μg RNA per sample was used as input material for RNA sample preparations. Sequencing libraries were generated using an RNA Library Prep Kit for Illumina (NEB, Ipswich, MA, USA). The library preparations were sequenced on a HiSeq X Ten platform (Illumina, San Diego, CA, USA) and paired-end reads were generated. In total, 18 libraries were sequenced for the two cultivars, both of which were sampled at 131 DAF, 238 DAF and 302 DAF.

### Sequence and expression analysis

The adaptor sequences and low-quality sequence reads were removed from the data sets. Raw sequences were transformed into clean reads after data processing. Tophat2 software tool [33] was used to map the clean reads with the reference genome for *C. sinensis* [34]. The quantification of gene expression levels was estimated by the fragments per kilobase of transcripts per million fragments mapped (FPKM). The differential expression analysis was performed using the DESeq R package [35] based on the read count for each gene across the fruit developmental stages. The resulting *P* values were adjusted using Benjamini and Hochberg’s approaches for controlling the false discovery rate (FDR). The relative expression levels of each transcript calculated by DESeq were assigned as differentially expressed genes (DEGs; fold change >2 and FDR < 0.01). Pearson’s correlation coefficients were determined among the three biological replicates for each sample to determine the reliability of the DEGs. Relative expression levels for each transcript were calculated based on the _lg_FPKM, and a heat map was generated using the ‘pheatmap’ software (https://cran.r-project.org/web/packages/pheatmap/index.html).

### Cluster analysis and gene annotation

The change in expression patterns for each transcript in *C. sinensis* were characterized by K-means clustering during fruit development. All statistical analyses were performed in R version 2.15.3. The clustering of transcription patterns based on FPKM levels was performed using the K-means method with the Euclidean similarity metric. The Blast2go software [36] was used to perform the gene ontology functional classification (biological process, molecular function and cellular component) for all DEGs. Kyoto Encyclopedia of Genes and Genomes (KEGG) pathway analysis was performed using KOBAS 2.0 to test the statistical enrichment [37]. Only the differentially expressed genes involved in the KEGG pathway with an adjusted q-value <0.05 found by KOBAS were assigned to biological pathways with statistically significant, other pathways (q-value >0.05) were ignored.

### Protein preparations for each sample

Fruit flesh for each cultivar, with three independent replicates, collected at 302 DAF were ground in liquid nitrogen into a powder. Proteins were extracted using a previously published protocol, and the resulting proteins were digested by trypsin and labeled using a Tandem Mass Tag (TMT) as previously described [38]. Then, the tryptic peptides were fractionated by high pH reverse-phase HPLC using an Agilent 300 Extend C18 column.

### LC-MS/MS analysis

LC-MS/MS analysis was performed according to previously described protocols [39]. The tryptic peptides were separated using an EASY-nLC 1000 UPLC system (Thermo Fisher Scitific, Waltham, MA, USA) and were subjected to a nanospray ionization source followed by MS/MS in a Q Exactive Plus (Thermo Fisher Scitific, Waltham, MA, USA) coupled to a UPLC. Peptides were selected for MS/MS using a normalized collision energy setting of 30, and ion fragments were detected in an Orbitrap at a resolution of 17,500. The data-dependent procedure alternated between a single MS scan followed by 20 MS/MS scans with 15.0 s dynamic exclusions. The automatic gain control was set at 5E4, and the fixed first mass was set as 100 *m*/*z*.

### Database search

The resulting MS/MS data were processed using the Maxquant search engine (v.1.5.2.8). Tandem mass spectra were searched using amino acid sequences corresponding to the genes in an annotated *C. sinensis* genome (http://citrus.hzau.edu.cn/orange/download/index.php). Trypsin/P was specified as the cleavage enzyme and allowed up to two missing cleavages. The mass tolerance for the precursor ions was set as 20 ppm in the ‘First search’ and 5 ppm in the ‘Main search’, and the mass tolerance for fragment ions was set as 0.02 Da. The FDR was adjusted to <1% and the minimum score for peptides was set >40.

### Bioinformatics analysis

The KEGG database was used to identify enriched pathways using a two-tailed Fisher’s exact test to examine the enrichment of the differentially expressed proteins against all identified proteins (*P* value <0.05). All significantly changed proteins and their cognate mRNA were enriched by the KEGG pathway based on the direction of the change. Hierarchical clustering based on the enrichment was further visualized using a heat map constructed with the “heatmap.2” function from the “gplots” R-package.

### Real time quantitative RT-PCR validation

Total RNAs from blood orange fruit flesh were isolated using an RNA isolation kit (Huayueyang, Beijing, China). cDNA synthesis was performed using the PrimeScript first-strand cDNA synthesis kit (Takara, Dalian, China). The relative transcription levels of a selected group of identified DEGs (at each fruit development stage) were reexamined by real-time quantitative RT-PCR as previously described [40]. *Elongation factor* was used as the internal control. The primer sequences (Additional file 9) for the selected genes were designed with the Premier Primer software or were previously published [4, 17, 41, 42]. All qPCR reactions were performed on a Roche480 (Roche, Basel, Switzerland). The reactions were performed in a reaction volume of 20 μL with five replicates for each sample. Error bars on each column indicate standard error (SE) from five independent replicates. The capital letter above each error bar means statistically significant in the relative expression level between three fruit developmental period (*P* < 0.01). Statistical differences in relative expression of each gene between different developmental periods were evaluated using an ANOVA by SPSS (IBM, Armonk, NY, USA).

### Statistical analysis

Statistical differences in fruit quality between the two varieties were evaluated using an ANOVA by SPSS (IBM, Armonk, NY, USA). Pearson’s correlation coefficients were determined independently for relative expression folds of each gene and fruit-quality parameter with SPSS. Data were considered to be significant when *P* < 0.05.

## Additional files

Additional file 1: The measurements of TSS/TA ratio during fruit ripening. (TIFF 225 kb)

Additional file 2: Sequencing data and mapped reads by RNA-seq. (XLSX 12 kb)

Additional file 3: The Pearson’s correlation analysis among three biological replicates and Venn diagram of the DEGs in comparison with cultivars during fruit ripening. (A) Heat map of Pearson’s correlation coefficients. (B) Venn diagram of differential expression genes. (TIFF 790 kb)

Additional file 4: GO classifications of DEGs for blood orange in comparison with fruit developmental stage to stage. (TIFF 4416 kb)

Additional file 5: Comparison of expression ratios from transcriptomic (y-axis) and proteomic (x-axis) profiling. Log_2_expression ratios of transcript or protein were calculated between two cultivars. Significant changes of DEGs in two expression levels are color-coded: blue, only proteins in significant expression; green, transcripts; red, both. (A) Concordance test between expression changes in 4248 pairs of mRNA and cognate protein. (B) Pearson’s correlations analysis of the different pairs of mRNA and cognate protein. (TIFF 3073 kb)

Additional file 6: Analysis of co-expression of proteins and cognate mRNA identified at fruit delayed-harvest stage between two cultivars. (XLSX 478 kb)

Additional file 7: Important genes involved into the anthocyanin biosynthesis at fruit delayed-harvest stage with transcription and protein at significant expression levels. (TIFF 1511 kb)

Additional file 8: Important genes involved into the sucrose and citrate metabolism at fruit-delayed harvest stage with transcription and protein at expression levels. (TIFF 1377 kb)

Additional file 9: The pairs of primers used to amplify the targeting genes by qPCR (XLSX 11 kb)

## Abbreviations

ABA: Abscissic acid
DEGs: Differential expression genes
GO: Gene ontology
KEGG: Kyoto Encyclopedia of Genes and Genomes
LC-MS/MS: Liquid chromatogram-tandem mass spectrometry
TA: Titratable acid
TMT: Tandem Mass Tag
TSS: Fotal soluble solids
